# Bioactive Lignan Honokiol Alleviates Ovarian Oxidative Stress in Aging Laying Chickens by Regulating SIRT3/AMPK Pathway

**DOI:** 10.3390/antiox13030377

**Published:** 2024-03-19

**Authors:** Yiqiu Chen, Zhaoyu Yang, Jingchun Bai, Xinyu Wang, Qiongyu Yuan, Yuling Mi, Caiqiao Zhang

**Affiliations:** Department of Veterinary Medicine, College of Animal Sciences, Zhejiang University, Hangzhou 310058, China; 22117122@zju.edu.cn (Y.C.); 12117050@zju.edu.cn (Z.Y.); 22117036@zju.edu.cn (J.B.); 22217098@zju.edu.cn (X.W.); qiongyu@zju.edu.cn (Q.Y.); yulingmi@zju.edu.cn (Y.M.)

**Keywords:** Honokiol, ovarian aging, oxidative stress, Sirtuins, AMPK, chicken

## Abstract

Aging is not only a key internal cause of age-related diseases in humans but also poses a threat to the productivity of farm animals with longer breeding cycles, such as laying chickens. Various measures were taken to prolong the laying period by reducing oxidative stress to improve poultry ovarian functions. Within the mitochondria, SIRT3, a member of the Sirtuin family, plays an important role in post-translational modifications and the regulation of protein activities involved in energy metabolism and oxidative response. This study aimed to investigate the alleviating effect of a bioactive lignan Honokiol (HKL) on oxidative stress in aging chicken ovaries in order to retard decline in egg production. The results showed that HKL treatment restored the abnormal balance between cell proliferation and apoptosis, and it enhanced the antioxidant capacity of the H_2_O_2_-induced small white follicles (SWFs) by activating the SIRT3/AMPK pathway. Moreover, HKL significantly increased total egg production, the number of yellow follicles, and the mRNA expression of yolk synthesis and deposition-related genes, serum estrogen, and antioxidant levels. These findings suggest that HKL holds promise in enhancing the egg productivity of aging laying chickens by promoting yolk deposition and reducing ovarian oxidative stress.

## 1. Introduction

The ovaries of female animals exhibit aging symptoms much earlier than other organs in the body, marked by a decline in the number and quality of oocytes. Ovarian aging is characterized by hormonal changes and diminished fertility. Ovarian hormones impact several target tissues, including the brain, bones, and reproductive system, thereby influencing general health [1]. Follicles are functional units of the female reproductive lifespan, with interactions between oocytes, granulosa cells (GCs), and theca cells (TCs) to regulate follicle development and atresia [2].The egg-laying performance of poultry primarily depends on the growth and development level of follicles. After 580 days of age, commercial laying chickens typically undergo accelerated ovarian aging, experiencing slowed follicle formation and development alongside increased follicular atresia, resulting in low productivity and breeding value [3]. In the atretic follicles (AFs), notable changes occur predominantly in GCs, with cell apoptosis observed earlier in GCs than in oocytes and theca cells [4]. Compared with peak-laying chickens (D280), the apoptosis of GCs in aging chickens (D580) intensifies, accompanied by reduced yolk deposition in follicles and decreased endoplasmic reticulum–mitochondrial contact points in the ultrastructure of follicular cells [5,6]. Therefore, exploring ways to inhibit follicular atresia and improve oocyte quality will provide fundamental solutions for delaying ovarian aging and enhancing egg production performance in laying chickens.

Cellular senescence is considered the foundation of aging, which refers to the non-proliferative (cell cycle arrest) but viable cellular state triggered by stress signals and physiological processes [7]. These stressors include genotoxic substances, nutrient deficiencies, hypoxia, mitochondrial dysfunction, and oncogene activation, triggering senescence subroutines involving DNA damage, mitochondrial damage, and oxidative stress damage [8]. Over time, senescent cells persist in producing cytokines and inflammatory mediators, thereby affecting the surrounding microenvironment and fostering chronic inflammation and fibrosis [9]. The accumulation of senescent cells within tissues diminishes the tissue regeneration capacity, leading to organ dysfunction and subsequently leading to aging [10]. There are twelve hallmarks of aging in three categories: main causes (genomic instability, telomere attrition, etc.), stress causes (deregulated nutrient-sensing, mitochondrial dysfunction, and cellular senescence), and comprehensive causes (altered intercellular communication, chronic inflammation, etc.) [11]. The forthcoming challenge is to improve our understanding of their interaction network between the hallmarks and to develop corresponding effective strategies.

The free radical theory stands as a cornerstone in explaining the mechanism of aging. When exposed to exogenous or endogenous factors, reactive oxygen species (ROS) accumulate and spread, initiating peroxidation reactions within cellular components. This disturbance results in an imbalance of oxidative stress damage and repair, which ultimately leads to cell death, aging, or carcinogenesis [12]. Mitochondria serve dual roles as the energy converters of cells and the main source of ROS [13]. As aging progresses, mitochondrial function deteriorates due to multiple mechanisms, including the accumulation of mitochondrial DNA mutations, insufficient protein homeostasis, and alterations in mitochondrial dynamics [11]. Senescent cells often exhibit mitochondrial dysfunction, characterized by reduced respiratory chain transfer capacity. This dysfunction is evidenced by decreased membrane potential, heightened electron leakage, and impaired rates of fusion and division [14]. Although the number of mitochondria in senescent cells may increase, their ability to produce ATP is weakened [15]. Studies have shown that, in aging animals, glycolysis and anaerobic metabolism increases, while aerobic metabolism decreases, and the efficiency of ATP generation significantly decreases [16]. Mitochondrial dysfunction not only impairs the contribution of mitochondria to energy metabolism but also enhances the production of ROS, triggering inflammation, aging phenotypes, and cell death [17]. Despite the longstanding focus on the association between mitochondrial dysfunction and aging, unraveling its exact mechanism remains a major challenge in aging research. In addition, due to the abundance of mitochondria in oocytes, metabolic damage caused by mitochondrial dysfunction holds particular significance in ovarian aging [18].

Furthermore, based on the free radical theory, antioxidants represent the largest class of compounds in reproductive aging research. As women age, there is an increase in reactive oxygen species (ROS) within oocytes, accompanied by a decline in antioxidant levels, resulting in diminished quantity and quality of oocytes [19]. Various antioxidants, such as N-acetyl-L-cysteine (NAC) [20], flavonoids [21], vitamins C and E [22], and coenzyme Q10 (CoQ10) [23], have been experimentally proven to clear excess ROS and enhance the antioxidant capacity in rodent models. The longevity effects of antioxidants in the female reproductive system include the preservation of ovarian reserve, augmentation of primordial and healthy follicle numbers, reduction of atretic follicle proportions, increase in litter size, and enhancement of estrous cycle regularity.

Honokiol (HKL), a bioactive lignan phytochemical derived from the traditional Chinese medicine Houpu (*Magnolia officinalis*), holds promise in combating oxidative stress and mitochondrial damage through activation of SIRT3, primarily located in mitochondria. By harnessing this mechanism, HKL effectively curtails excessive ROS production, mitigates mitochondrial damage, and attenuates cell death [24]. Moreover, HKL can restore SIRT3 expression during acute injury, increase AMPK activity, inhibit mitochondrial fragmentation and apoptosis, and reduce mitochondrial damage and oxidative stress [25]. Despite these promising attributes, there is little theoretical research on ovarian aging and its application in livestock and poultry production by HKL.

This study aimed to explore the protective effect of HKL on the development of ovarian follicles in aging chickens through in vitro and in vivo experiments. Specifically, we investigated the effects of oxidative stress on follicular development, encompassing phenomena such as cell cycle arrest, heightened apoptosis, weakened antioxidant capacity, and the downregulation of the SIRT3/AMPK pathway. In addition, our inquiry extends to elucidating the alleviating effect of HKL on oxidative stress damage in aging ovaries. Through our endeavors, we aspire to furnish evidence for the role of HKL in resisting follicle development retardation and mitigating ovarian aging in aging chickens.

## 2. Materials and Methods

### 2.1. Experimental Animals and Materials

Hyline white chickens (*Gallus domesticus*) were purchased and grown on a nearby commercial farm (Huajie Poultry Company, Hangzhou, China). During the breeding process, the temperature and humidity were maintained at approximately 28–32 °C and 60%, respectively. The photoperiods were 16 h light and 8 h dark.

Blood samples were collected from the wing vein; follicular tissues were collected from the small white follicle (SWFs) (diameter 2–4 mm) of D280 (280-day-old peaking laying chickens) and D580 (580-day-old aging chickens). Additionally, all levels of follicles were separated and counted, especially atretic follicles (AFs) and prehierarchical follicles (PHFs)—white follicles (WFs) and yellow follicles (YFs). Ovaries and PHFs were also weighed.

### 2.2. Culture of SWFs and Treatments of Chemicals

For in vitro experiments, every SWF was cultured in the 24-well plate containing 500 μL DMEM complete medium (Hyclone, Logan, UT, USA), supplemented with 5% fetal calf serum (FCS, Tauranga, New Zealand), 100 IU/mL penicillin, and 100 μg/mL streptomycin (Hyclone, Logan, UT, USA), along with 10 μg/mL insulin, 5 μg/mL transferrin, and 30 nM selenite (ITS, Sigma-Aldrich, St. Louis, MA, USA). The cultures were maintained at 38.5 °C with 5% CO_2_.

SWFs were preincubated with 1 mM H_2_O_2_ (Sinopharm Chemical Reagent, Shanghai, China) for 24 h and treated with Honokiol (HKL, Meilun Bio-Technology, Shanghai, China) for 48 h, as previously reported [26]. The optimal concentration of HKL for subsequent research was selected from different concentrations (0, 0.1, 1, 10, and 100 μM). In the follicular oxidative stress model, SWFs were randomly assigned to 4 groups: control, 1 mM H_2_O_2_, 1 mM H_2_O_2_ + 10 μM HKL, and 10 μM HKL. The SIRT3 inhibitor 3-TYP and AMPK inhibitor Compound C (CC) were obtained from MedChemExpress (MCE, Monmouth Junction, NJ, USA). All the chemicals were initially dissolved in DMSO for reserve solution kept at −20 °C and then redissolved in DMEM medium before use. SWFs were treated with 50 μM 3-TYP or 10 μM CC, respectively. In the pathway inhibition models, SWFs were also randomly assigned to 6 groups: H_2_O_2_, H_2_O_2_ + HKL, H_2_O_2_ + HKL + 3-TYP or CC, and 3-TYP or CC. Furthermore, SWFs were cultured in a complete medium mixed with 10 μg/mL BrdU (Sigma-Aldrich, St. Louis, MA, USA) for the final 24 h to conduct the BrdU incorporation test. Following treatment, SWFs were collected and fixed in 4% paraformaldehyde for staining and morphological observation. Some of the SWFs were used for biochemical analysis and qRT-PCR.

### 2.3. Animal Experiment

For in vivo experiments, 30 D580 chickens (1.5–2 kg BW) were randomly assigned to the corresponding control and HKL-feeding group (2 experimental groups × 3 replicates/group × 5 chickens/replicate), respectively. HKL powder was mixed with purified water to form a 20 mg/mL suspension. Each chicken in the HKL group was fed with HKL at a dose of 10 mg/kg for 14 consecutive days. The control group was assigned 1 mL of pure water. The egg production of each group was recorded every day. On the last day, 3 chickens were chosen randomly from each replicate group for morphological and biochemical analyses.

### 2.4. Staining and Morphological Observation

Hematoxylin and eosin (H&E) staining: Follicular tissues were preserved in 4% paraformaldehyde (PFA) at 4 °C for 24–48 h, followed by dehydration in graded ethanol and xylene, embedding in paraffin, and sectioning at a thickness of 5 μm. H&E staining was carried out in compliance with standard procedures. Eclipse 80i microscope (Nikon, Tokyo, Japan) was used for morphological observation. For every group, at least three follicular tissues were dissected into slices; and for each tissue slice, at least three fields of view were observed.

Immunofluorescence (IF) staining: IF staining was performed following the conventional methods. Primary antibodies used in the IF experiments included mouse anti-BrdU monoclonal antibody (1:200, G3G4, DSHB, Iowa City, IA, USA) and rabbit anti-PCNA monoclonal antibody (1:100, ET1605-38, HuaBio, Hangzhou, China). The secondary antibodies were TRITC-conjugated goat anti-mouse antibody (1:100, BS11502, Bioworld Technology, Bloomington, MN, USA) and TRITC-conjugated goat anti-rabbit antibody (1:50, HA1016, HuaBio, Hangzhou, China). DAPI counterstaining was adopted to observe the cell nuclei. The fluorescence images (magnification at 20×) were photographed using an IX70 fluorescence microscope (Olympus, Tokyo, Japan). BrdU-positive cells were appeared red, and the nuclei appeared blue.

TUNEL assay: Following the manufacturer’s instructions, the BrightGreen Apoptosis Detection kit (TUNEL, Vazyme, Nanjing, China) was used to perform the TUNEL assay. Cell nuclei were counterstained with DAPI. The fluorescence images were captured by an IX70 fluorescence microscope (Olympus, Tokyo, Japan). TUNEL-positive cells appeared green, and the nuclei presented as blue. The fluorescence intensity was determined using ImageJ software.

### 2.5. Biochemical Analysis

Measurements of antioxidant capacity: After homogenization, centrifugation, dilution, and other sample preprocessing of the in vitro experimental follicular tissue or in vivo experimental serum, the following antioxidant capacity and cellular energy metabolism indicators were measured: malonaldehyde (MDA), glutathione (GSH), catalase (CAT), total superoxide dismutase (SOD), total antioxidant capacity (T-AOC), adenosine triphosphate (ATP), and nicotinamide adenine dinucleotide (NAD^+^), according to the kit instructions (Nanjing Jiancheng Institute of Bioengineering, Nanjing, China).

Determination of estrogen and liver parameters: The levels of estradiol (E_2_) and progesterone (P_4_) in serum samples were measured by Abbott’s chemiluminescence kits (Abbott, Longford, Ireland), and the contents of albumin (ALB), globulin (GLB), aspartate aminotransferase (AST), and γ-glutamyl transpeptidase (GGT) of serum samples were detected by the biochemical kits (Nanjing Jiancheng Institute of Bioengineering, Nanjing, China).

### 2.6. RNA Extraction and qRT-PCR

Total RNA was extracted from SWFs, using TRIzol reagent (Takara, Shiga, Japan). A NanoDrop 2000c (Thermo Scientific, Waltham, MA, USA) was used to quantify the amount of RNA present. The cDNA was synthesized using a HiScript II 1st Strand cDNA Synthesis Kit (Vazyme, Nanjing, China), following the manufacturer’s instructions. The resulting cDNA was employed in qRT-PCR analysis as a corresponding template. Using the SYBR Premix Ex Taq kit (Vazyme, Nanjing, China) and the ABI 7500HT real-time PCR equipment (Applied Biosystems, Foster City, CA, USA), the qRT-PCR was conducted with the recommended conditions and protocols in the manual. The relative levels of mRNA expression were determined using the β-actin-standardized 2^−ΔΔCt^ formula method. A list of PCR primers is presented in Table 1.

### 2.7. Western Blot

The SWFs were homogenized using 400 μL of RIPA lysis buffer (P0013B, Beyotime, Nanjing, China) supplemented with proteinase inhibitor PMSF (Solarbio, Beijing, China). Total protein concentrations were determined using a BCA protein assay kit (Nanjing Jiancheng Institute of Bioengineering, Nanjing, China). Subsequently, 20 μg of total protein was mixed with SDS 5×loading buffer and RIPA to a final volume of 30 μL, heated at 95 °C for 15 min, separated on 10% SDS-PAGE gel, and transferred to a polyvinylidene difluoride (PVDF) membrane (0.22 μm, Millipore, Bedford, MA, USA). The membrane was then blocked with 5% skim milk at room temperature for 1 h, followed by overnight incubation at 4 °C with the corresponding primary antibodies, including rabbit anti-SIRT3 (1:1000, R1511-3), mouse anti-β-actin (1:10,000, EM21002, HuaBio, Hangzhou, China), rabbit anti-AMPKα1/AMPKα2 (1:1000, A17290), and rabbit anti-phospho-AMPKα1/AMPKα2 (1:1000, AP1171, Abclonal, Wuhan, China). On the following day, the PVDF membrane was incubated at room temperature for 1 h with corresponding secondary antibodies, including HRP-conjugated goat anti-rabbit IgG (1:50,000, HA1001) or HRP-conjugated goat anti-mouse IgG (1:50,000, HA1006, HuaBio, Hangzhou, China). Using an enhanced chemiluminescence kit (Bio-Rad, Hercules, CA, USA), immunological signals were detected by a ChemiScope 3400 Mini device (Clinx, Shanghai, China). The expression levels of proteins were quantified using ImageJ v2.3.0 software.

### 2.8. Statistical Analysis

All experiments were repeated at least 3 times, and results were presented as mean ± standard error of the mean (SEM). Data were analyzed by one-way analysis of variance (ANOVA) with Tukey’s multiple comparisons test or Student’s *t*-test, using GraphPad Prism 9.0 software. Statistical significance was presented as *p* < 0.05.

## 3. Results

### 3.1. Alleviating Effect of HKL on Imbalance of Cell Proliferation and Apoptosis in the H_2_O_2_-Induced SWFs

**Figure 1:**
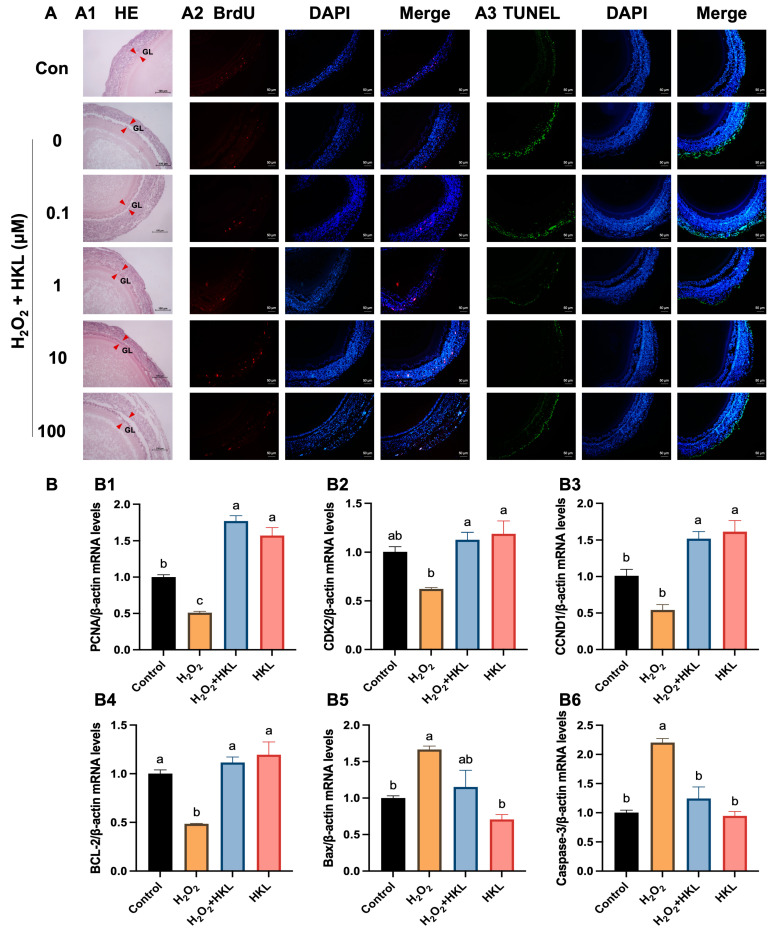
Effect of HKL on cell proliferation and apoptosis in the H_2_O_2_-induced SWFs. (**A**) (**A1**) H&E staining. (**A2**) IF staining. BrdU-positive cells (red) for evaluating cell proliferation. (**A3**) TUNEL assay. TUNEL-positive cells (green) for evaluating cell apoptosis. Nuclei were stained with DAPI (blue). GL (red arrowheads): granulosa layer. Scale bar: 100 μm or 50 μm. (**B**) The mRNA expression of proliferation-related genes ((**B1**–**B3**) *PCNA*, *CDK2*, and *CCND1*) and apoptosis-related genes ((**B4**–**B6**) *BCL-2*, *Bax*, and Caspase-3) in SWFs. Data are expressed as mean ± SEM (*n* = 4). Different lowercase letters indicate significant differences (*p* < 0.05). The expression results are relative to the control group level.

The H&E staining results showed that treatment of 0.1 μM and 10 μM HKL can restore the oxidative damage inflicted on SWFs by H_2_O_2_ to the level of the control group, which is mainly manifested in the tight structure and the regular morphology of the granulosa cells (Figure 1A(A1)). According to the results of BrdU immunofluorescence staining, H_2_O_2_ treatment significantly inhibited cell proliferation of SWFs, while the addition of 1 μM and 10 μM HKL significantly increased the BrdU positivity rate of SWFs, reaching levels comparable to the control group (Figure 1A(A2)). Additionally, the TUNEL assay results demonstrated a notable increase in cell apoptosis in SWFs induced by H_2_O_2_. Remarkably, treatment with 10 μM HKL significantly attenuated this increase in cell apoptosis, restoring it to the same level as in the control group (Figure 1A(A3)). Based on the effects of different doses of HKL on tissue morphology, cell proliferation, and apoptosis of D280 SWFs in vitro, 10 μM HKL could alleviate the morphological damage of follicular tissue induced by H_2_O_2_ and emerged as the optimal treatment concentration for the subsequent experiments.

Furthermore, qRT-PCR experiments revealed that exposure to H_2_O_2_ significantly downregulated the expression levels of proliferation-related genes and upregulated the expression levels of apoptotic genes. In the H_2_O_2_-induced group, there was a notable reduction in the expression of *PCNA*, *CDK2*, *CCND1,* and *BCL-2* mRNAs, while the expression of *Bax* and Caspase-3 mRNAs was significantly increased compared with the control group (Figure 1B). In the H_2_O_2_ + HKL treatment group, the expressions of *PCNA*, *CDK2*, and *CCND1* were significantly higher than those in the H_2_O_2_-induced group and control group, while the levels of *BCL-2*, *Bax*, and Caspase-3 were restored to the control levels (Figure 1B).

### 3.2. Activation of HKL on Antioxidant Capacity and SIRT3/AMPK Pathway in the H_2_O_2_-Induced SWFs

**Figure 2:**
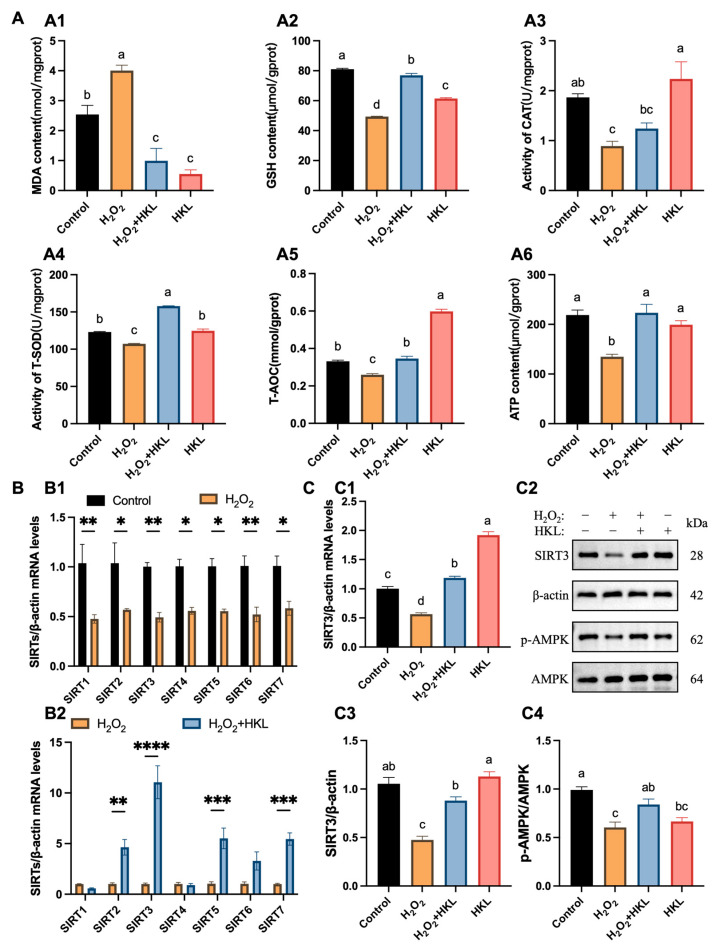
Effect of HKL on antioxidant capacity and SIRT3/AMPK pathway in the H_2_O_2_-induced SWFs. (**A**) Determination of antioxidant capacity and energy metabolism ((**A1**–**A6**) MDA, GSH, CAT, T-SOD, T-AOC, and ATP) in the H_2_O_2_-induced SWFs (*n* = 4). (**B**) Relative expression of *SIRTs* mRNA after H_2_O_2_ (**B1**) or HKL (**B2**) treatment in SWFs (*n* = 3). (**C**) Relative expression of *SIRT3* mRNA (**C1**), SIRT3, and pAMPK proteins (**C2**–**C4**) after different treatments in SWFs (*n* = 3). Data are expressed as mean ± SEM. Different lowercase letters indicate significant differences (*p* < 0.05); * *p* < 0.05, ** *p* < 0.01, *** *p* < 0.001, and **** *p* < 0.0001. The expression results are relative to the control group level.

In terms of oxidative stress biochemical indicators, the measurement results found that H_2_O_2_ decreased tissue antioxidant capacity, resulting in oxidative stress damage in SWFs. Specifically, compared with the control group, the H_2_O_2_-induced group exhibited a significant increase in the content of MDA, while experiencing a significant decrease in the content of GSH, as well as in the enzymatic activities of CAT and T-SOD, along with reductions in T-AOC and ATP content (Figure 2A). Conversely, the MDA content in the H_2_O_2_ + HKL group was significantly lower than that in the H_2_O_2_-induced group and the control group. Meanwhile, the GSH content, T-SOD activity, and T-AOC and ATP content in the H_2_O_2_ + HKL group significantly increased, compared with the H_2_O_2_-induced group (Figure 2A).

Additionally, qRT-PCR experiments showed that treatment with H_2_O_2_ significantly reduced the expression of *SIRT1–SIRT7* genes with a similar trend (Figure 2B(B1)). Meanwhile, upon supplementation with HKL in H_2_O_2_-induced SWFs, variations in the expression of *SIRT1–SIRT7* genes were observed, with the most significant increase in *SIRT3* expression (Figure 2B(B2)). This suggests that HKL may alleviate the decreased expression level of *SIRTs* family genes induced by H_2_O_2_ oxidative stress through a SIRT3-dependent manner. According to Western blot quantification analysis, after H_2_O_2_ treatment, the expression of *SIRT3* mRNA, SIRT3 protein, and AMPK phosphorylation in SWFs significantly decreased (Figure 2C). Notably, treatment of H_2_O_2_ + HKL significantly restored the expression of SIRT3 mRNA and protein, as well as the phosphorylation level of AMPK, elevating them to levels comparable to those observed in the control group. However, the addition of HKL alone had no significant effect on SIRT3 protein expression, compared with the control group (Figure 2C).

### 3.3. The Suppression of SIRT3 Inhibitor on Antioxidant Effect of HKL

**Figure 3:**
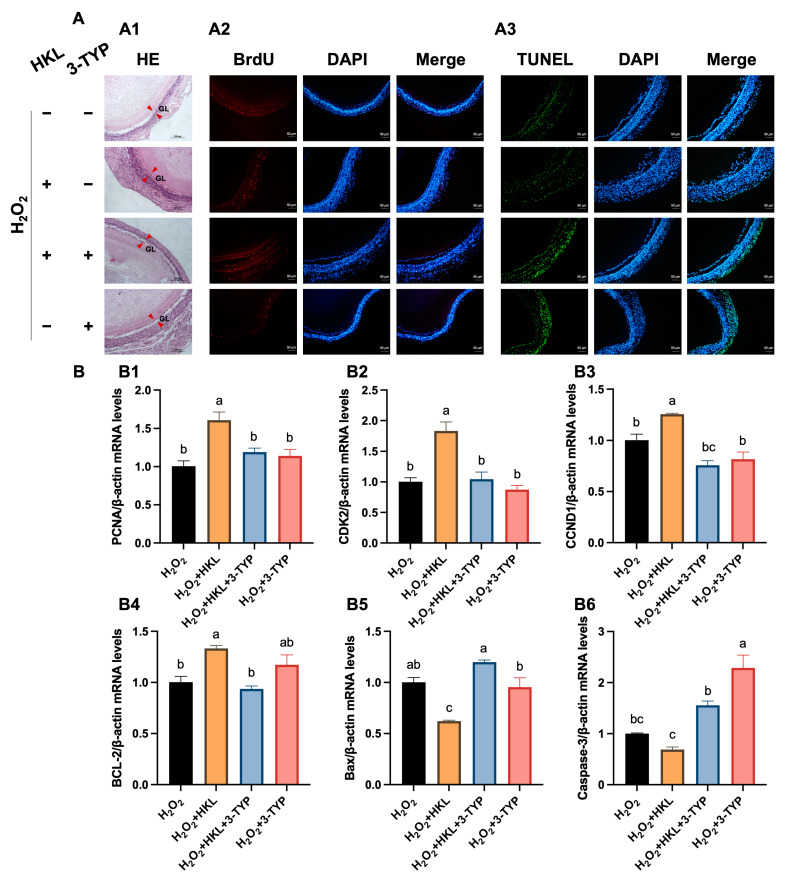
The suppression of 3-TYP on the promotion of cell proliferation and inhibition of cell apoptosis by HKL. (**A**) (**A1**) H&E staining and (**A2**) IF staining. BrdU-positive cells (red) for evaluating cell proliferation. (**A3**) TUNEL assay. TUNEL-positive cells (green) for evaluating cell apoptosis. Nuclei were stained with DAPI (blue). GL (red arrowheads): granulosa layer. Scale bar: 100 μm or 50 μm. (**B**) Effect of different treatments on the expression of proliferation-related genes ((**B1**–**B3**) *PCNA*, *CDK2*, and *CCND1*) and apoptosis-related genes ((**B4**–**B6**) *BCL-2*, *Bax*, and Caspase-3) in SWFs (*n* = 4). Different lowercase letters indicate significant differences (*p* < 0.05). The expression results are relative to the H_2_O_2_ group level.

To further explore whether the alleviation effect of HKL on H_2_O_2_-induced oxidative stress in SWFs is achieved by activating the SIRT3/AMPK pathway, 3-TYP was selected as the SIRT3 inhibitor. The H&E staining results revealed that HKL could alleviate the morphological damage inflicted upon the granulosa layer of follicular tissue induced by H_2_O_2_. However, when 3-TYP was administered, it countered this alleviating effect, leading to similar damage as the H_2_O_2_-induced group (Figure 3A(A1)). In addition, the results of BrdU immunofluorescence staining exhibited that HKL could increase the cell proliferation rate of H_2_O_2_-induced SWFs, while 3-TYP offset this increased effect, which was as low as the level of the H_2_O_2_-induced group (Figure 3A(A2)). Similarly, the TUNEL assay results found that HKL could reduce the apoptosis rate of SWFs induced by H_2_O_2_. In contrast, the administration of 3-TYP promoted apoptosis of SWFs, with levels even significantly higher than the level in the H_2_O_2_-induced group (Figure 3A(A3)).

Moreover, the qRT-PCR analysis demonstrated that the inhibitor 3-TYP offset the upregulating effect of HKL on the expression of proliferation-related genes and the downregulating effect of HKL on the expression of apoptosis-related genes. Specifically, compared with the H_2_O_2_-induced group, the expression levels of *PCNA*, *CDK2*, *CCND1,* and *BCL-2* mRNAs in the H_2_O_2_ + HKL group were significantly increased. However, with co-treatment of HKL and 3-TYP, the expression levels of those proliferation-related genes in H_2_O_2_-induced SWFs were not significantly different from those in the H_2_O_2_-induced group; but the expression levels of those apoptosis-related genes showed significant differences, indicating a high level of apoptosis (Figure 3B).

**Figure 4:**
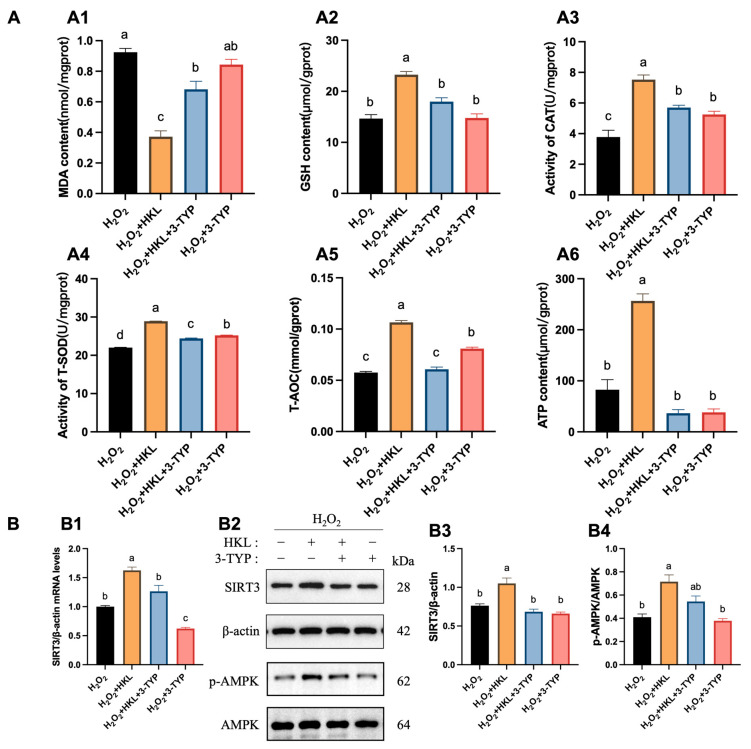
The suppression of 3-TYP upon the promotion of antioxidant capacity and activation of the SIRT3/AMPK pathway by HKL. (**A**) Determination of antioxidant capacity and energy metabolism ((**A1**–**A6**) MDA, GSH, CAT, T-SOD, T-AOC, and ATP) in the H_2_O_2_-induced SWFs after 3-TYP treatment (*n* = 4). (**B**) Relative expression of *SIRTs* mRNA (**B1**) (*n* = 4); relative expression of SIRT3 and pAMPK proteins (**B2**–**B4**) in SWFs (*n* = 3). Data are expressed as mean ± SEM. Different lowercase letters indicate significant differences (*p* < 0.05).

Likewise, 3-TYP eliminated the effect of HKL on improving the total antioxidant capacity and energy metabolism level of tissues. Compared with the H_2_O_2_-induced group, the H_2_O_2_ + HKL group exhibited a significant reduction in the MDA content, along with significant increases in the GSH content, CAT activity, T-SOD activity, and T-AOC and ATP content. However, compared with the H_2_O_2_ + HKL group, when 3-TYP was added alone or co-treated, the MDA content was significantly increased, while the GSH content, CAT activity, T-SOD activity, and T-AOC and ATP content were significantly reduced (Figure 4A). Similarly, treatment with 3-TYP reversed the upregulating effect of HKL on H_2_O_2_-induced SIRT3 protein expression and AMPK phosphorylation in SWFs (Figure 4B).

### 3.4. The Suppression of AMPK Inhibitor on Antioxidant Effect of HKL

**Figure 5:**
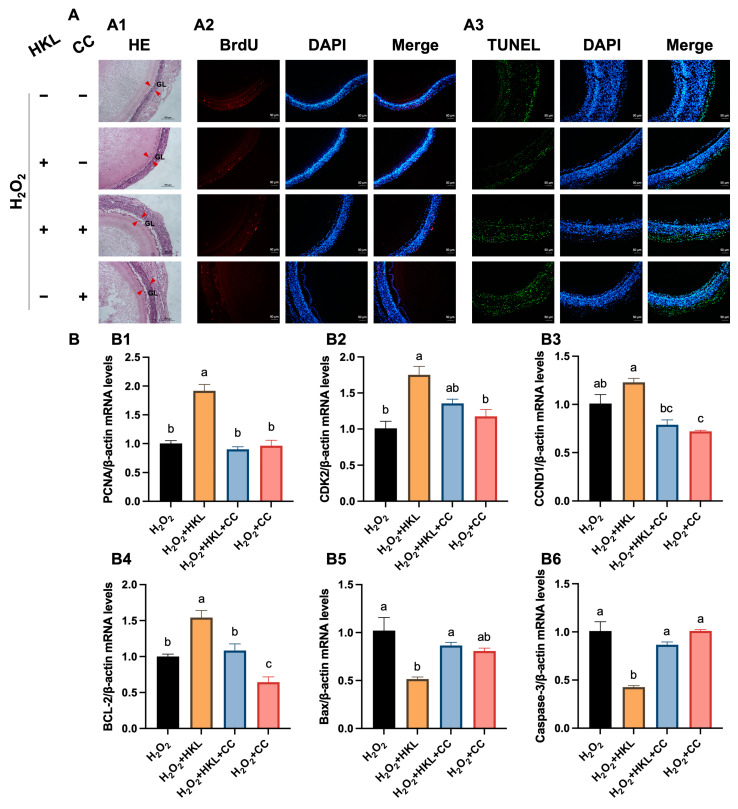
The suppression of CC on promotion of cell proliferation and inhibition of cell apoptosis by HKL. (**A**) (**A1**) H&E staining. (**A2**) IF staining. BrdU-positive cells (red) for evaluating cell proliferation. (**A3**) TUNEL assay. TUNEL-positive cells (green) for evaluating cell apoptosis. Nuclei were stained with DAPI (blue). GL (red arrowheads): granulosa layer. Scale bar: 100 μm or 50 μm. (**B**) Effect of different treatments on the expression of proliferation-related genes ((**B1**–**B3**) *PCNA*, *CDK2*, and *CCND1*) and apoptosis-related genes ((**B4**–**B6**) *BCL-2*, *Bax*, and Caspase-3) in SWFs (*n* = 4). Data are expressed as mean ± SEM. Different lowercase letters indicate significant differences (*p* < 0.05). The expression results are relative to the H_2_O_2_ group level.

We then utilized CC as an AMPK pathway inhibitor to determine the target of HKL for alleviating oxidative stress. Initially, the H&E staining results indicated that the addition of inhibitor CC reversed the alleviating effect of HKL on morphological damage in H_2_O_2_-induced SWFs (Figure 5A(A1)). Subsequently, SWFs exposed to HKL and CC exhibited a significantly lower cell proliferation rate and higher cell apoptosis rate, compared with the H_2_O_2_ + HKL group (Figure 5A). Meanwhile, compared with the H_2_O_2_ + HKL group, lower expression levels of *CCND1* and higher expression levels of *Bax* and Caspase-3 were detected in SWFs from the H_2_O_2_ + HKL + CC group, while *PCNA*, *CDK2,* and *BCL-2* showed no significant alterations. This suggests that the addition of inhibitor CC mainly inhibited the downregulation of HKL on the expression of apoptosis-related genes in follicle tissue induced by H_2_O_2_ (Figure 5B).

**Figure 6:**
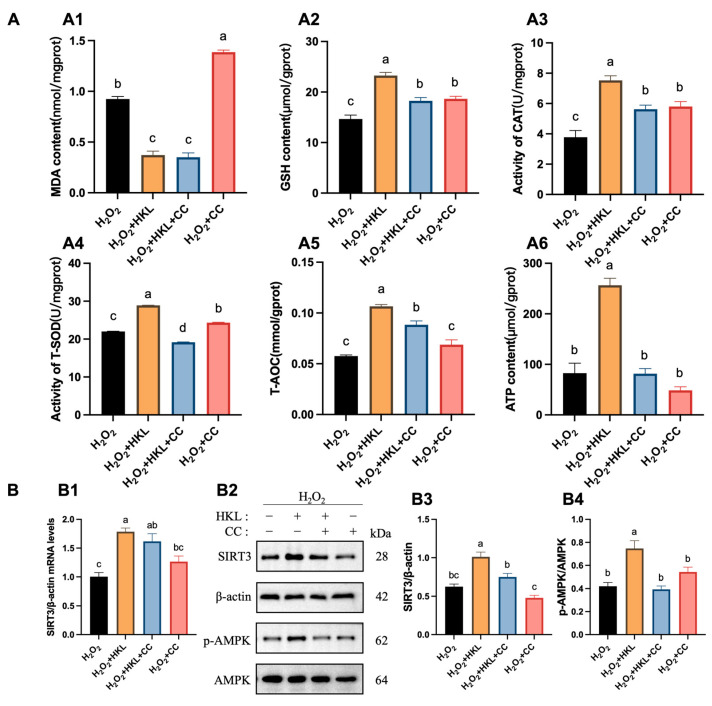
The suppression of CC on promotion of antioxidant capacity and activation of SIRT3/AMPK pathway by HKL. (**A**) Determination of antioxidant capacity and energy metabolism ((**A1**–**A6**) MDA, GSH, CAT, T-SOD, T-AOC, and ATP) in the H_2_O_2_-induced SWFs after CC treatment (*n* = 4). (**B**) Relative expression of *SIRT3* mRNA (**B1**) (*n* = 4); relative expression of SIRT3 and pAMPK proteins (**B2**–**B4**) in SWFs (*n* = 3). Data are expressed as mean ± SEM. Different lowercase letters indicate significant differences (*p* < 0.05).

In terms of antioxidant capacity and energy metabolism function, after the corresponding treatment of the inhibitor, H_2_O_2_-induced SWFs treated with HKL and CC had less GSH content, CAT activity, T-SOD activity, and T-AOC and ATP content (Figure 6A). This confirms that inhibiting the AMPK pathway weakens the protective effect of HKL on H_2_O_2_-induced oxidative stress in SWFs. In addition, significantly lower levels of SIRT3 and p-AMPK protein expression were detected in H_2_O_2_-induced SWFs with HKL and CC treatment, in contrast to the H_2_O_2_ + HKL group (Figure 6B).

### 3.5. Effects of HKL on Egg Production and Follicle Development of Aging Chickens

**Figure 7:**
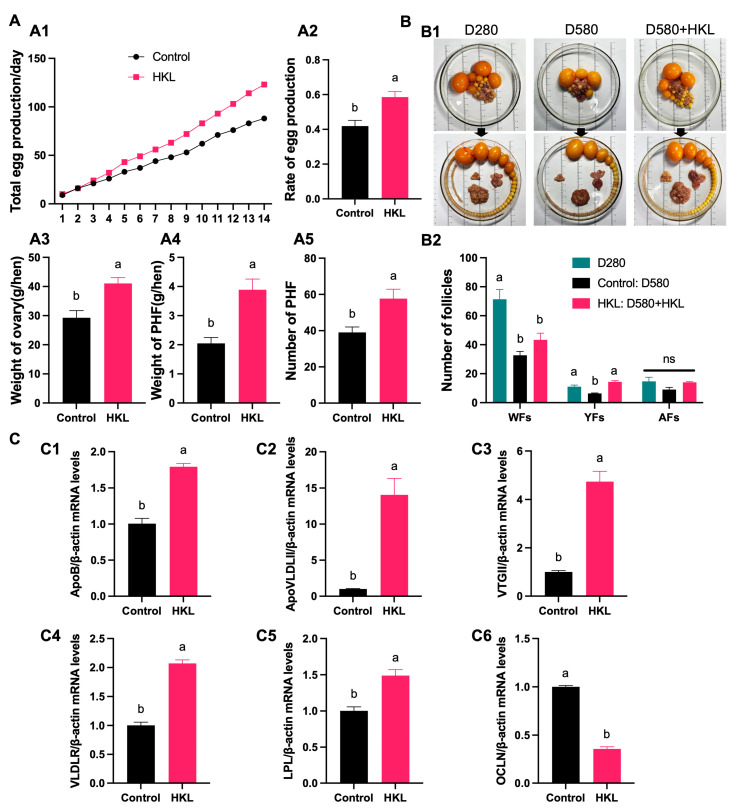
Effect of HKL on laying performance and ovarian development of D580 chickens. (**A**) Effect of feeding HKL on egg production and ovarian development: (**A1**,**A2**) total egg production, egg production rate; (**A3**,**A4**) weight of ovary and PHFs; and (**A5**) number of PHFs (*n* = 4). (**B**) Hierarchical development in D280, D580, and the HKL-fed D580 group: (**B1**) Follicles are arranged in order; (**B2**) the number of different follicles (n = 4). (**C**) The mRNA expression of yolk synthesis-related genes ((**C1**–**C3**) *ApoB*, *ApoVLDLII*, and *VTGII*) in D580 livers and yolk deposition-related genes ((**C4**–**C6**) *VLDLR*, *LPL*, and *OCLN*) in D580 SWFs (*n* = 4). Data are expressed as mean ± SEM. Different lowercase letters indicate significant differences (*p* < 0.05). ns: not significant. The expression results are relative to the control group level.

To verify whether feeding HKL can improve the egg production performance of aging chickens and alleviate ovarian aging, an in vivo experiment was conducted by gavage-feeding HKL to 580-day-old naturally aging chickens (D580) at a dose of 10 mg/kg BW for 14 consecutive days. Based on daily counts, the total egg production and egg production rate were calculated (Figure 7A(A1)). The statistical results showed that, compared with the control group, the egg production rate of the HKL group increased by 37.5% (Figure 7A(A2)). Meanwhile, the HKL group also demonstrated a significant increase in the ovary weight and PHF weight and number (Figure 7A(A3–A5)).

We further counted all levels of PHFs and added the group of D280 chickens as a reference. The quantity statistics revealed that, compared with the young group, the number of white follicles (WFs) and yellow follicles (YFs) in the aging control group exhibited a significant reduction, while the number of atretic follicles (AFs) displayed no significant change. This indicates that, during the aging process, the ability of the ovaries to produce primordial follicles is markedly reduced. A comparison between the aging control group and the aging HKL group showed that HKL significantly increased the number of YFs, while the number of WFs did not change significantly (Figure 7B).

The expression levels of genes associated with yolk precursor synthesis in liver tissue and yolk deposition-related genes in ovarian tissue of D580 chickens in the feeding experiment were detected by qRT-PCR. The transcription analysis found that, compared with the control group, the expression of *ApoB*, *ApoVLDLII,* and *VTGII* in the liver of the HKL group was significantly increased (Figure 7C(C1–C3)). Meanwhile, the expression levels of *VLDLR* and *LPL* mRNAs in the SWFs of the HKL group were significantly increased, while the expression levels of *OCLN* were notably reduced (Figure 7C(C4–C6)).

### 3.6. Effects of HKL on Serum Biochemical Indicators of Aging Chickens

**Figure 8:**
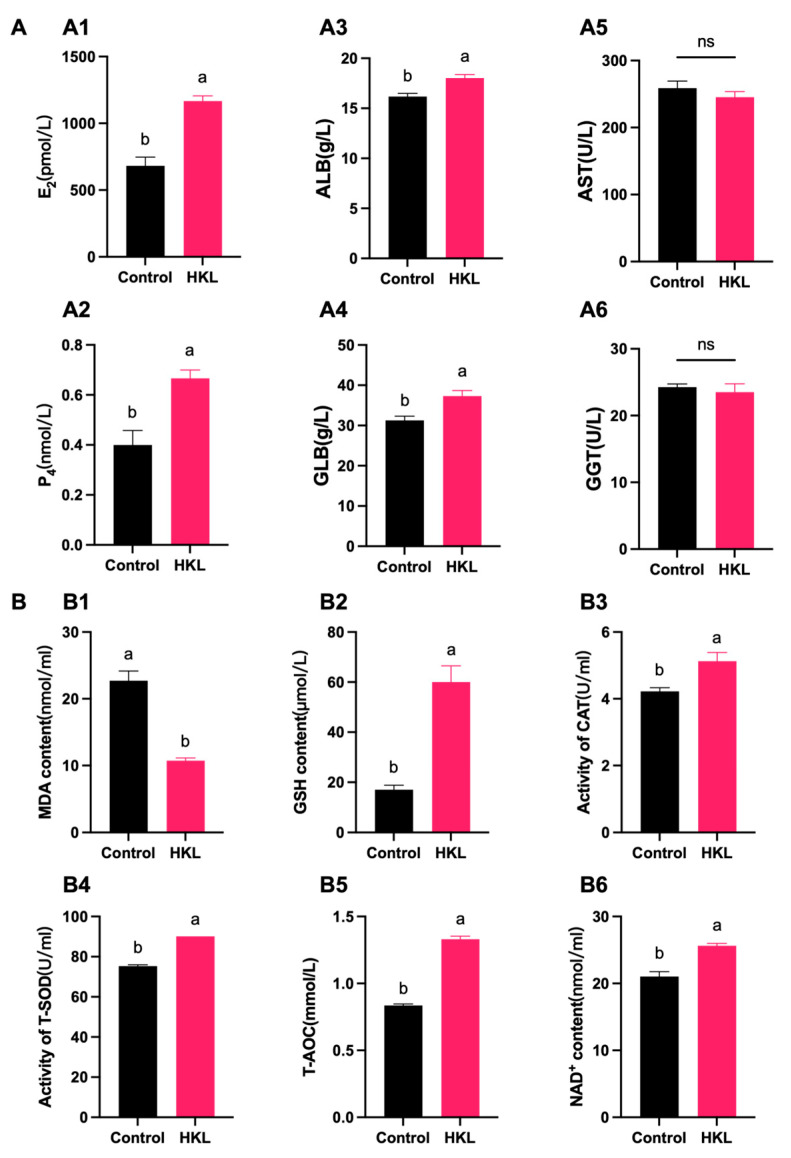
Effects of HKL on serum estrogen, liver function, and antioxidant levels in D580 chickens. (**A**) Effect of HKL on estrogen and liver indicators of D580 serum in vivo. E_2_ (**A1**) and P_4_ (**A2**) levels were determined by immunological methods in serum. ALB (**A3**) and GLB (**A4**) contents, and AST (**A5**) and GGT (**A6**) enzyme activities were determined by biochemical methods in serum (*n* = 4). (**B**) Determination of antioxidant capacity and energy metabolism ((**B1**–**B6**) MDA, GSH, CAT, T-SOD, T-AOC, and NAD^+^) in serum (*n* = 4). Data are expressed as mean ± SEM. Different lowercase letters indicate significant differences (*p* < 0.05).

The biochemical analysis found that, compared with the control group, the E_2_ and P_4_ levels of aging chickens were significantly increased after HKL treatment, indicating that HKL enhanced the estrogen secretion ability of aging chickens (Figure 8A(A1,A2)). The content of ALB and GLB significantly increased, indicating that HKL augmented the protein synthesis ability of aging chickens (Figure 8A(A3,A4)). However, there were no significant changes in AST and GGT enzyme activities, indicating that HKL had no significant effect on the liver detoxification and metabolic function of aging chickens (Figure 8A(A5,A6)).

According to the measurement of oxidative stress biochemical indicators, compared with the control group, the MDA content of serum samples of the HKL group exhibited a significant reduction. Conversely, there was a notable increase in GSH content, as well as in CAT and T-SOD activities, along with elevated levels of T-AOC and NAD^+^ contents (Figure 8B).

### 3.7. Activation of SIRT3/AMPK Pathway by HKL Feeding in SWFs

**Figure 9:**
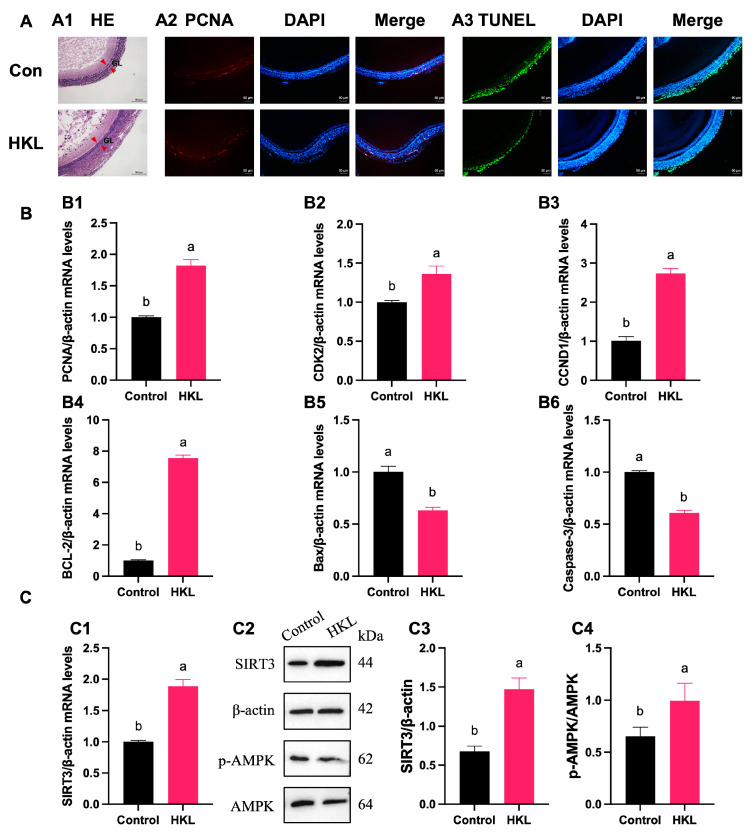
Effect of HKL on the proliferation and apoptosis of SWFs and SIRT3/AMPK pathway in D580 chickens. (**A**) (**A1**) H&E staining. (**A2**) IF staining. PCNA-positive cells (red) for evaluating cell proliferation. (**A3**) TUNEL assay. TUNEL-positive cells (green) for evaluating cell apoptosis. Nuclei were stained with DAPI (blue). GL (red arrowheads): granulosa layer. Scale bar: 100 μm or 50 μm. (**B**) The mRNA expression of proliferation-related genes ((**B1**–**B3**) *PCNA*, *CDK2*, and *CCND1*) and apoptosis-related genes ((**B4**–**B6**) *BCL-2*, *Bax*, and Caspase-3) in SWFs (*n* = 4). (**C**) The expression levels of *SIRT3* mRNA (**C1**) (*n* = 4), SIRT3 and pAMPK proteins (**C2**–**C4**) in SWFs (*n* = 3). Data are expressed as mean ± SEM. Different lowercase letters indicate significant differences (*p* < 0.05).

The H&E staining results showed that the continuous feeding of HKL for 14 days had an alleviating effect on the morphological damage of SWFs in aging chickens, mainly characterized by a denser structure of the granulosa layer (Figure 9A(A1)). Additionally, feeding HKL can visibly increase the number of BrdU-positive cells and reduce the number of TUNEL-positive cells in SWFs (Figure 9A(A2,A3)). The balance between cell proliferation and apoptosis was corroborated by the qRT-PCR analysis, which demonstrated a significant increase in the expression of *PCNA*, *CDK2*, *CCND1,* and *BCL-2* mRNAs, coupled with a marked decrease in the expression of *Bax* and Caspase-3 mRNAs (Figure 9B).

The results from qRT-PCR experiments revealed a notable increase in the expression level of *SIRT3* mRNA in aging chicken ovaries of the HKL group compared with the control group (Figure 9C(C1)). Furthermore, protein quantitative analysis also confirmed the increased expression of SIRT3 protein and AMPK phosphorylation in aging chicken ovaries of the HKL group (Figure 9C(C2–C4)).

## 4. Discussion

The laying cycle of chickens can be divided into early laying period, peak laying period, and late laying period. When laying chickens reach 28 weeks of age, they enter the peak laying period, lasting for 20 weeks or more. After 54 weeks of age, they enter the late laying period, with a decrease in egg production rate and breeding value [27]. In general, farms will regularly discard laying chickens over 580 days old, indicating the end of their reproductive lifespan. Therefore, the selection of D280 and D580 chickens as peak laying chickens and aging chickens, respectively, in this study aligns with the typical reproductive lifespan of laying chickens in commercial farms. High-concentration H_2_O_2_ treatment has been widely used to establish exogenous oxidative stress models, which may weaken antioxidant capacity [28]. We used the H_2_O_2_-induced D280 SWFs established in our previous laboratory as the model to investigate the effect of HKL on reducing induced oxidative stress in follicles [5]. In this study, the significant increase in MDA content and decrease in antioxidant capacity observed in SWFs of the H_2_O_2_ treatment group confirm the establishment of oxidative stress conditions. However, treatment with HKL effectively alleviated the decrease in antioxidant capacity induced by H_2_O_2_, as evidenced by the increased activity of antioxidants such as CAT, total SOD, and T-AOC, along with elevated GSH content. These findings suggest that HKL alleviates the decrease in antioxidant capacity induced by H_2_O_2_ by increasing the activity of these antioxidants in SWFs. These antioxidant substances can effectively clear ROS and maintain the redox balance. HKL also improved the decline in the antioxidant status of D580 aging chickens.

Cell cycle arrest, or the cessation of cell proliferation, is a characteristic of cellular senescence that may be a warning response to harmful stimuli or abnormal proliferation [8]. In addition, defective cellular quality control systems lead to inflame-aging through the accumulation of intracellular waste, such as damaged mitochondria, protein aggregates, and lipofuscin particles [29]. DNA damage and senescence-associated secretion phenotype (SASP) dominated by inflammatory cytokines, growth factors, and proteases induce cell apoptosis in aging cells [30]. Furthermore, follicular atresia arises from heightened apoptotic processes regulated by apoptotic factors [31]. Active Caspase-3 plays a crucial role in the cascade response of the apoptosis signaling pathway and serves as a common downstream effector shared by multiple apoptosis pathways. The Bcl-2 family, located on the membranes of different organelles, regulates caspase enzyme activity. Among its members, Bcl-2, Bcl-XL, and Bcl-W are anti-apoptotic—they bind to or degrade pro-apoptotic proteins to prevent cell apoptosis. Conversely, Bax, Bad, and Bid are pro-apoptotic—they are involved in transducing certain cell death signals [32]. According to the qRT-PCR data, H_2_O_2_ treatment resulted in a reduction in the expression of cell cycle marker genes such as *PCNA*, *CDK2*, and *CCND1*, as well as the anti-apoptotic gene BCL-2, accompanied by an increase in the expression of pro-apoptotic genes *Bax* and Caspase-3. This was consistent with the finding that the labeling rate of BrdU-positive cells significantly decreased, and TUNEL-positive cells significantly increased in the fluorescence experiment. These findings collectively indicate a decrease in cell proliferation and an increase in cell apoptosis in the H_2_O_2_-induced D280 SWFs. Additionally, HKL treatment reversed these changes caused by H_2_O_2_, indicating that HKL has a regulatory effect on the homeostasis of SWFs cell proliferation and apoptosis. Remarkably, similar effects were observed in D580 chickens fed with HKL.

The two nutritional sensors AMPK and Sirtuins are key regulators in cellular energy metabolism, emitting signals of nutrient deficiency and catabolism, respectively. Their activation may mediate lifespan extension [33]. AMPK activity is closely related to mitochondrial physiology. Upon activation, AMPK phosphorylates PGC1α, thereby inducing mitochondrial gene expression and regulating mitochondrial quality [34]. Furthermore, reduced AMPK activity may contribute to mitochondrial dysfunction and weakened dynamics during aging [35]. The Sirtuin family (including SIRT1–SIRT7) is a class of deacetylase enzymes with a conserved core region, using nicotinamide adenine dinucleotide (NAD^+^) as a substrate [36]. Sirtuins play an important role in post-translation modification (PTMs) and activity regulation of proteins. They can interact with proteins such as P53, FOXO, PGC-1α, NF-κB, and Ku70 to regulate cellular stress responses, thereby affecting biological processes such as cell metabolism, aging, and apoptosis [37]. Notably, SIRT3, predominantly localized in mitochondria, regulates ATP levels, and loss of SIRT3 leads to reduced ATP levels in multiple organs [11]. Activation of SIRT3 is also implicated in the extension of caloric restriction lifespan by deacetylating mitochondrial proteins and activating mitochondrial superoxide dismutase 2 (SOD2) and isocitrate dehydrogenase 2 (IDH2), thereby mitigating DNA damage caused by oxidative stress [38,39]. In addition, SIRT1 [40] and SIRT3 [41] can participate in a positive feedback loop with AMPK. In this study, we found that HKL not only alleviated oxidative stress but also increased the expression of Sirtuins genes, especially SIRT3, in H_2_O_2_-induced SWFs, along with an increase in the expression of SIRT3 protein and AMPK phosphorylation levels. Moreover, when inhibiting SIRT3 and AMPK, HKL could not effectively alleviate oxidative stress. Therefore, we propose that the antioxidant effect of HKL depends on the SIRT3/AMPK pathway.

ATP is the basic carrier of energy conversion in living organisms, and its content changes directly affect energy metabolism and cell function. Usually, when cells undergo apoptosis, necrosis, or adverse stress, the ATP level decreases, suggesting impaired or decreased mitochondrial function [42]. In the elderly, heightened oxidative stress disrupts the coupling between oxygen consumption and ATP synthesis, impairing the recycling of AMP through energy-regulated kinases and diminishing ATP synthesis [43]. Consequently, ATP availability declines with age, accompanied by reduced efficiency of energy generation through OXPHOS, prompting increased reliance on aerobic glycolysis and anaerobic metabolism to partially compensate for decreased ATP synthesis [43]. Our findings demonstrate that, while improving antioxidant status, HKL can restore the ATP content of H_2_O_2_-induced SWFs.

NAD^+^ is a key coenzyme and an important enzyme substrate for redox reactions and energy metabolism naturally synthesized in the body. Systemic decline in NAD^+^ is considered a key molecular event that occurs during the aging process and oxidative stress [44]. Therefore, supplementing NAD^+^ holds significant promise in delaying aging and relieving age-related diseases [45]. Evidence suggests that the increase in NAD^+^ can restore hallmarks of mitochondrial dysfunction during aging, increase energy metabolism [46], and maintain glucose homeostasis [47]. Studies on mice have shown that supplementation with NAD^+^ reduced ROS levels, improved ovarian mitochondrial metabolism [48], restored oocyte vitality, and enhanced fertility [49]. In addition, some antioxidant therapies, such as coenzyme Q10, vitamins C and E, and flavonoids, have been shown to increase NAD^+^ levels and improve mitochondrial function through the SIRT3-dependent pathway to alleviate ovarian aging [50]. In this study, feeding HKL significantly increased the NAD^+^ content in the serum of aging chickens, reversing the decrease in NAD^+^ during aging.

A prolonged reproductive lifespan is indicated by a constantly expanding follicle pool. The irreversible decline in follicle quantity and quality will lead to deterioration of ovarian function. This decline manifests as dysfunction of follicle generation and hierarchical development, as well as a decrease in ovarian steroidogenesis [51]. Follicles are regulated by hormones from the hypothalamic–pituitary–ovarian axis, governing ovulation cycles and the duration of the reproductive lifespan. Estrogen levels in female animals decline with age, and the low estrogen content in the microenvironment accelerates aging. Granulosa cells produce estrogens, mainly estradiol (E_2_); and theca cells produce progesterone (P_4_) and androgens [51]. Estrogen is a crucial hormone that controls the female reproductive system, preserves bone metabolism, and affects the cardiovascular system and cognitive function [52]. Following ovulation, the corpus luteum releases progesterone, which is involved in nerve regeneration, neuroprotection, and preserving pregnancy [53]. In this study, continuous feeding of HKL for two weeks resulted in a significant increase in serum E_2_ and P_4_ levels, as well as an increase in the number of follicles and an improvement in the follicular development of aging chickens.

The liver–blood–ovary axis regulates egg production. Apart from its primary function in lipid synthesis, the liver serves as the assembly site for yolk precursors. The main components of yolk precursors are small particles of very low density lipoprotein (VLDLy) and vitellogenin (VTG), and their production is controlled by estradiol, E_2_. E_2_, produced by the ovary, is transported to the liver via the bloodstream, where it interacts with estrogen receptors (ER-α and ER-β) on the liver to regulate the synthesis of Vitellogenin 2 (VTGII), Apolipoprotein B (ApoB), and very low density apolipoprotein II (ApoVLDLII). This process ultimately facilitates the synthesis of yolk precursors [54]. In addition, during yolk deposition, fat metabolism is active in the ovary, characterized by high expression levels of lipoprotein lipase (LPL) and low expression levels of Occludin (OCLN) [55]. In this study, feeding HKL significantly upregulated the expression levels of yolk precursor synthesis-related genes in the liver and genes associated with yolk deposition in follicles of aging chickens, indicating that HKL can effectively stimulate the formation of yellow follicles in vivo.

## 5. Conclusions

According to the findings of this study, HKL demonstrates the capability to mitigate the adverse effects of H_2_O_2_-induced oxidative stress, including the inhibition of cell proliferation and promotion of apoptosis, in SWFs. This is achieved by enhancing antioxidant capacity through the activation of the SIRT3/AMPK pathway, thereby preventing follicle atresia and ovarian quality decline. Furthermore, HKL supplementation leads to increased estrogen levels, improved yolk deposition efficiency, and promoted hierarchical development and egg production performance in aging laying chickens. In summary, this study suggests that HKL can be an effective measure to delay follicular atresia or ovarian aging in laying chickens by regulating antioxidant capacity and improving energy metabolism through activating the SIRT3/AMPK pathway to counteract oxidative stress.

## Figures and Tables

**Table 1:** Sequences of the PCR primers.

Gene Name	Accession No.	Primer Sequence (5′–3′)	Product Size (bp)
*PCNA*	NM_204170.2	F: GGGCGTCAACCTAAACAGCA R: AGCCAACGTATCCGCATTGT	97
*CDK2*	NM_001199857.1	F: TCCGTATCTTCCGCACGTTG R: GCTTGTTGGGATCGTAGTGC	183
*CCND1*	NM_205381.1	F: CCTCAAGAAAAGCCGGTTGC R: CTGCGGTCAGAGGAATCGTT	86
*BCL-2*	NM_205339.2	F: ATCGTCGCCTTCTTCGAGTT R: ATCCCATCCTCCGTTGTCCT	150
*Bax*	XM_015290060.2	F: GGATGACAGGAAAGTACGGCA R: TCACCAGGAAGACAGCGTAT	173
Caspase-3	NM_204725.1	F: CAGCTGAAGGCTCCTGGTTT R: GCCACTCTGCGATTTACACG	98
*SIRT1*	NM_001004767.2	F: CCCCGCAGCCCGATAAC R: ATACGTGGTCTTGGGGTCCA	127
*SIRT2*	NM_001017414.2	F: GTGACGCCCCGTCCTATC R: TTCCGCAGCAGCTCCATATC	104
*SIRT3*	NM_001199493.2	F: AGACCCAACTACGCCCACTA R: GAGGGATCCCAGCAACTCG	109
*SIRT4*	XM_025155741.3	F: GATTTTGTGCACCAACGCCT R: GCCAGTGCAAACCTGCATAG	75
*SIRT5*	NM_001276364.2	F: TCGATGCACCAACTGTGGAA R: CTGGATCTGGAGCCCCTTTC	86
*SIRT6*	NM_001039320.2	F: ACAACAATGAGCTCTCCGGT R: GTGGATCGAAAATCTCGGGGA	113
*SIRT7*	NM_001291971.1	F: CGGCAGGAGGAGGTATGTGA R: CGGTAGTCTGGGATCGAAGC	140
*ApoB*	NM_001044633.2	F: GCGGTACAAGCAGAAGGTGT R: AGACGTCGCTGGTCAGAATC	88
*ApoVLDLII*	NM_205483.3	F: CACCACTGTCCCTGAAGTGC R: CATCAGGGATGACCAGCCAG	115
*VTGII*	NM_001031276.2	F: CTTACCTCCTCAAGGTCCGC R: CCGGGTGAAACACGATGAGA	116
*VLDLR*	NM_205229.2	F: TCTGAGATGTGGAGGATTCAAC R: GAAGAACAGCCCAAGCTCCT	83
*LPL*	NM_205282.2	F: ACTGAAACTTTTTCGCCGCTG R: TTCATCTCAGCTTCGGGATCG	127
*OCLN*	NM_205128.1	F: GGCGGAGGGCCACCA R: GTCGTCCACGTAGTAGGAGC	137
β-actin	NM_205518	F: ACACCCACACCCCTGTGATGAA R: TGCTGCTGACACCTTCACCATTC	136

## Data Availability

All data analyzed are contained within the article.
